# Detection of Infectious Viruses Using CRISPR-Cas12-Based Assay

**DOI:** 10.3390/bios11090301

**Published:** 2021-08-28

**Authors:** Chandana S. Talwar, Kwang-Hyun Park, Woo-Chan Ahn, Yong-Sam Kim, Oh Seok Kwon, Dongeun Yong, Taejoon Kang, Euijeon Woo

**Affiliations:** 1 Disease Target Structure Research Center, Korea Research Institute of Bioscience and Biotechnology (KRIBB), 125 Gwahak-ro, Yuseong-gu, Daejeon 34141, Korea; chandanatalwar@kribb.re.kr (C.S.T.); ruuua@kribb.re.kr (K.-H.P.); wcahn@kribb.re.kr (W.-C.A.); 2 Department of Biomolecular Science, University of Science and Technology (UST), 217 Gajeong-ro, Yuseong-gu, Daejeon 34113, Korea; omsys1@kribb.re.kr (Y.-S.K.); oskwon79@kribb.re.kr (O.S.K.); 3 Genome Editing Research Center, Korea Research Institute of Bioscience and Biotechnology (KRIBB), 125 Gwahak-ro, Yuseong-gu, Daejeon 34141, Korea; 4 Infectious Disease Research Center, Korea Research Institute of Bioscience and Biotechnology (KRIBB), 125 Gwahak-ro, Yuseong-gu, Daejeon 34141, Korea; 5 Department of Laboratory Medicine and Research Institute of Bacterial Resistance, Yonsei University College of Medicine, 50-1 Yonsei-ro, Seodaemun-gu, Seoul 03722, Korea; deyong@yuhs.ac; 6 Bionanotechnology Research Center, Korea Research Institute of Bioscience and Biotechnology (KRIBB), 125 Gwahak-ro, Yuseong-gu, Daejeon 34141, Korea

**Keywords:** SARS-CoV-2, COVID-19, CRISPR-Cas12, virus, infectious disease

## Abstract

The outbreak of severe acute respiratory syndrome coronavirus 2 (SARS-CoV-2), which causes coronavirus disease-19 (COVID-19), has severely influenced public health and economics. For the detection of SARS-CoV-2, clustered regularly interspaced short palindromic repeats (CRISPR)-CRISPR associated protein (Cas)-based assays have been emerged because of their simplicity, sensitivity, specificity, and wide applicability. Herein, we have developed a CRISPR-Cas12-based assay for the detection of SARS-CoV-2. In the assay, the target amplicons are produced by isothermal reverse transcription recombinase polymerase amplification (RT-RPA) and recognized by a CRISPR-Cas12a/guide RNA (gRNA) complex that is coupled with the collateral cleavage activity of fluorophore-tagged probes, allowing either a fluorescent measurement or naked-eye detection on a lateral flow paper strip. This assay enables the sensitive detection of SARS-CoV-2 at a low concentration of 10 copies per sample. Moreover, the reliability of the method is verified by using nasal swabs and sputum of COVID-19 patients. We also proved that the current assay can be applied to other viruses, such as Middle East respiratory syndrome coronavirus (MERS-CoV) and severe acute respiratory syndrome coronavirus (SARS-CoV), with no major changes to the basic scheme of testing. It is anticipated that the CRISPR-Cas12-based assay has the potential to serve as a point-of-care testing (POCT) tool for a wide range of infectious viruses.

## 1. Introduction

Since the first appearance of severe acute respiratory syndrome coronavirus 2 (SARS-CoV-2) in December 2019, it has spread to more than 200 countries, infecting 190 million and causing 4 million deaths as of July 2021 [1,2,3]. Although the current pandemic disease has affected human beings for over a year, this is not the first time that public health has been put at risk by viral outbreaks. In the last century, we have been threatened by evolving viruses, including Middle East respiratory syndrome coronavirus (MERS-CoV), severe acute respiratory syndrome coronavirus (SARS-CoV), Ebola, and recurring annual influenza, with mortality rates of 43, 10, 50, and 0.2%, respectively [1,2,4,5,6]. As we learn from the present and previous viral outbreaks, the treatment and vaccination of the newly emerging viruses are difficult and inevitably take time. Therefore, it is critical to diagnose infectious viruses rapidly and accurately for the prevention of viral spread.

Diagnosis using quantitative real-time polymerase chain reaction (qRT-PCR) has played an important role during the coronavirus disease-19 (COVID-19) pandemic because of high clinical sensitivity and selectivity [7,8,9]. However, the technique is dependent on sophisticated instruments and the process is time-consuming; as a result, it cannot meet the growing demand for rapid testing [10]. Another diagnostic method for the detection of SARS-CoV-2 is based on immunoglobulin (IgM/IgG) antibodies [11]. The IgM antibodies are produced 4–10 days post-infection and IgG antibodies are produced after around 2 weeks. Consequently, a low level of antibodies in the sample at the early stage of infection may lead to false-negative detection [11,12]. Recently, rapid antigen tests for SARS-CoV-2 have been developed, however, they are still limited in sensitivity and selectivity [13,14]. In this regard, researchers across the world are working on the development of an easy-to-use rapid diagnostic method for SARS-CoV-2 detection.

The clustered regularly interspaced short palindromic repeats (CRISPR)-CRISPR associated protein (Cas) system is a well-known tool in the field of gene editing due to its ease of use, accuracy, and high efficiency [15]. In addition to gene editing, some of the CRISPR-Cas proteins are being used to develop molecular diagnostic tools by using the collateral activities of proteins [16]. Recent advances include the specific high-sensitivity enzymatic reporter unlocking (SHERLOCK) and DNA endonuclease-targeted CRISPR trans reporter (DETECTR) systems, where Cas13 and Cas12 proteins are, respectively, used to recognize a specific target, with trans-cleavage collateral activity against fluorophore- and quencher-tagged reporter molecules [16,17].

Both Cas13 and Cas12 proteins have been used extensively for the purpose of CRISPR-Cas-based molecular detection of SARS-CoV-2. Cas13 has an advantage over Cas12 in detecting RNA pathogens owing to its sensitivity and primary RNA detection function. However, protospacer adjacent motif (PAM) restriction of the Cas12 protein can be advantageous when differentiating between two sequences that are highly similar and mismatch is observed in the PAM region. Cas12 dependency on PAM can add another layer of specificity. Additionally, while using Cas13, an RNA reporter has to be used which is more vulnerable to degradation from RNase contamination and can result in false positives or higher background noise. Using RNA as a primary target can sometimes result in the problem of inaccessible targets, due to RNA secondary structures, leading to lower efficiency of Cas13 activity.

During the COVID-19 pandemic, the SHERLOCK and DETECTR-based systems were quickly adapted to detect the SARS-CoV-2 pathogen. Since then, the CRISPR-based COVID-19 detection system has advanced rapidly with several modifications [18]. Systems such as All-In-One Dual CRISPR-Cas12a (AIOD-CRISPR) and STOPcovid.v2 were developed to work as a single pot reaction, increasing the ease of handling the samples [19,20]. Several digital detection systems of SARS-CoV-2 were also developed by integrating digital-based detection and by coupling smartphones with the system [21,22]. Systems such as point-of-care (POC)-CRISPR, which work as compact automated devices, have brought the CRISPR-based detection system close to being a fully functional point-of-care testing (POCT) system [23]. However, to date, all the developed CRISPR-Cas-based detection systems have mainly focused on the detection of the SARS-CoV-2 pathogen, as it should be in the given situation. Other closely related pathogens like SARS-CoV and MERS-CoV are still prevalent [24,25]. In addition to detecting and differentiating SARS-CoV-2 from SARS-CoV and MERS-CoV, it is necessary to specifically detect these pathogens. In this way, an integrated system can be developed to detect all three prevalent respiratory pathogens.

Herein, we report a CRISPR-Cas12-based assay for SARS-CoV-2. Isothermal reverse transcription-RPA (RT-RPA) is employed to produce target viral amplicons and the CRISPR-Cas12a/guide RNA (gRNA) complex is used not only to recognize the amplicons but also to cleave the fluorophore-tagged probes. We carefully tested the collateral activities of three different Cas12 proteins and selected Lbcpf1 enzyme for the assay. In addition, two kinds of detection methods including fluorescence and lateral flow assay (LFA) were adopted, providing the detection limit of 10 copies per sample. Most importantly, the developed assay could diagnose 12 COVID-19 patients and eight healthy people accurately. Lastly, it is demonstrated that the assay can be used for the detection of MERS-CoV and SARS-CoV. We anticipate that the CRSIPR-Cas12-based assay can be useful for the diagnosis of various infectious viruses.

## 2. Results

### 2.1. CRISPR-Cas12-Based Detection of SARS-CoV-2

**Figure 1:**
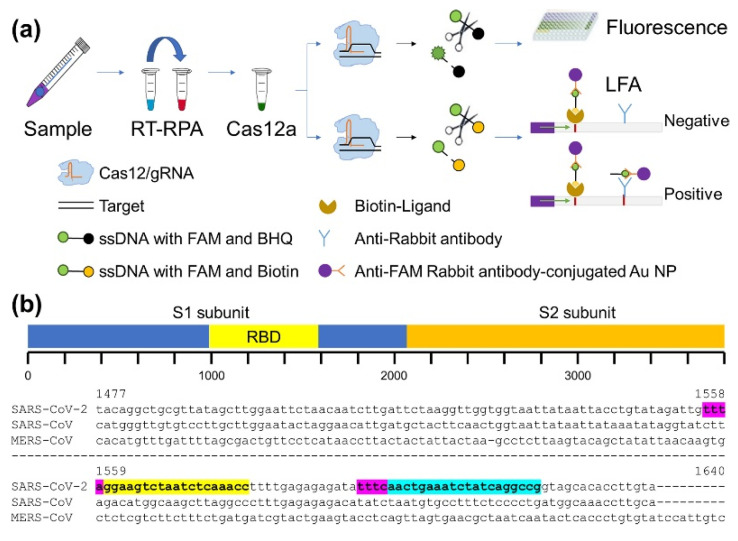
(**a**) Schematic representation of CRISPR-Cas12-based assay for SARS-CoV-2. (**b**) Sequence alignment of RBD region of S gene in SARS-CoV-2, SARS-CoV, and MERS-CoV. Regions colored in yellow and sky blue indicate SARS-CoV-2-specific T1 and T2, respectively, next to PAM sequence, marked in magenta.

The schematic illustration of the CRISPR-Cas12-based assay for SARS-CoV-2 is shown in Figure 1a. The assay includes the conversion of viral RNA to DNA and the following amplification using an RT-RPA method. Next, the amplified target is recognized by the Cas12/gRNA complex, leading to non-specific cleavage of the reporter DNA molecule and subsequent detection of the result in two ways. One is by conventional fluorescence detection, where the reporter molecule tagged with FAM and BHQ1 serves as the source of fluorescence after non-specific cleavage by the Cas12. The second mode of detection is lateral flow paper strip-based naked-eye detection. This is achieved using commercially available lateral flow strips designed to capture FAM- and biotin-tagged nucleic acids using anti-FAM rabbit antibody-conjugated Au nanoparticles (NPs), anti-rabbit antibody, and biotin ligand. In our assay, the uncleaved reporter molecules containing FAM-biotin were captured at the first detection line by the biotin ligand, which is considered the control band. Cleaved molecules were captured on the second detection line by an anti-rabbit antibody which serves as the test band.

For the accurate diagnosis of the virus, it is critical to select the target gene sequence precisely. To date, three highly pathogenic human coronaviruses have been identified, namely MERS-CoV, SARS-CoV, and SARS-CoV-2 [26]. In general, the entry of these viruses into the host system is facilitated by the interaction between the receptor on the host cell surface and the receptor-binding domain (RBD) in the spike protein of the virus coded by the S gene [27]. As SARS-CoV and SAR-CoV-2 share the same receptor (ACE2) on the surface of human cells [28], we carefully selected two targets of 20 nucleotide lengths located next to the PAM sequence (TTTN) by comparing the nucleotide sequence of the RBD region of SARS-CoV and SARS-CoV-2. These two targets are referred to as Target 1 (T1) and Target 2 (T2) (Figure 1b). Furthermore, we selected a positive control target next to the PAM in the POP7 gene of RNase P since RNase P has been used as a positive control in the analysis of clinical samples [29,30,31]. Thereafter, all samples were analyzed using two SARS-CoV-2-specific targets (T1 and T2) and the POP7 target as a control.

### 2.2. Optimization of Cas12 Proteins

**Figure 2:**
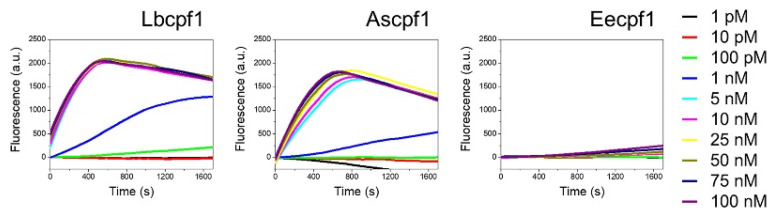
Collateral activity of Lbcpf1, Ascpf1, and Eecpf1 using varied concentrations of T1. Eecpf1 activity is measured in the presence of 1 mM MnCl_2_ compared to Lbcpf1 and Ascpf1 activities in MgCl_2_.

Cas12 proteins are reported to show collateral activities [15,17,32]. Upon the recognition of the primary target in a sequence-specific manner, they act on ssDNA molecules and cleave them non-specifically [33]]. We compared the collateral activities of three cpf1 enzymes (Lbcpf1, Ascpf1, and Eecpf1) using the synthesized DNA fragment of the S gene across concentrations ranging from 100 nM to 1 pM (Figure 2). The T1 region of the S gene was used as the target for this assay. The collateral activity was measured as a fluorescence read-out using a FAM-BHQ-tagged ssDNA reporter. As shown in Figure 2, Ascpf1 and Lbcpf1 provided highly comparable activities in the concentration range of 100 nM to 25 nM, whereas Eecpf1 did not show a significant increase even at the highest concentration of 100 nM. Among three Cas12 proteins, Lbcpf1 showed the highest activity at concentrations lower than 10 nM. According to previous reports, Eecpf1 showed increased fluorescence in the buffer condition where MgCl_2_ was replaced with 1 mM MnCl_2_ as a source of metal ions [33]. However, even in the presence of manganese, the collateral activity of Eecpf1 was significantly lower than that of Lbcpf1 or Ascpf1 across all the concentration ranges of the experiment. As a result, in this diagnostic assay, Lbcpf1 was selected and used for further analysis.

### 2.3. Detection of SARS-CoV-2

**Figure 3:**
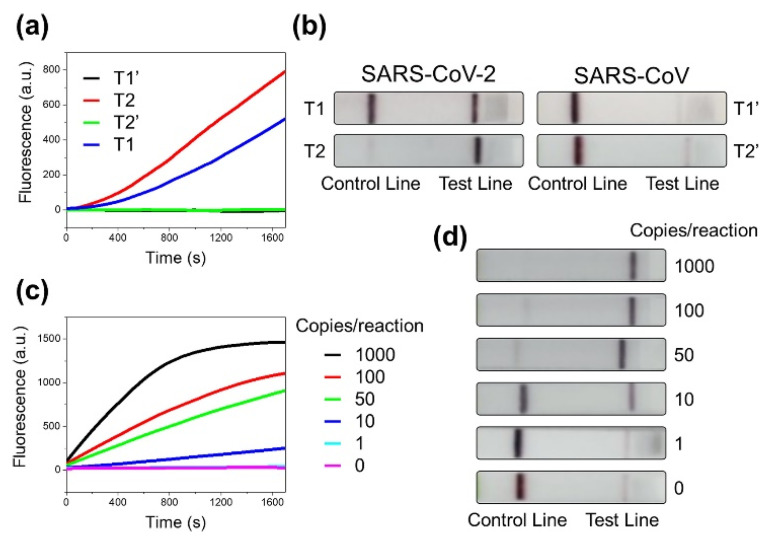
(**a**) Fluorescence and (**b**) LFA detection results of synthetic S gene of SARS-CoV-2 (T1 and T2) and SARS-CoV (T1′ and T2′) using CRISPR-Cas12-based assay. (**c**) Fluorescence and (**d**) LFA detection results of synthetic S gene of SARS-CoV-2 (T2) using CRISPR-Cas12-based assay. Copy number of T2 was varied from 1000 to 1 copy/reaction.

We carried out the detection of synthesized SARS-CoV-2 S gene (T1 and T2) using the CRISPR-Cas12-based assay. As shown in Figure 3a, the fluorescence results showed the efficient detection of both T1 and T2. These results were consistent with the results of LFA, where a significant band was observed at the test line for both T1 and T2 (Figure 3b). SARS-CoV, which shares 79% genetic similarity with SARS-CoV-2, might pose the problem of non-target recognition, thus leading to occasional false-positive results [34]. For the analysis of the specificity, we further tried to detect the synthesized S gene (T1′ and T2′) of SARS-CoV, keeping all reaction conditions the same. In the presence of the SARS-CoV S gene, no significant increase in signal was observed for either of the two targets, suggesting the specific recognition of SARS-CoV-2 without non-target effects towards SARS-CoV. The lateral flow strips also showed no band at the test line in the presence of the SARS-CoV S gene. Taken together, the fluorescence and LFA results indicate that our assay is capable of specifically detecting the SARS-CoV-2 target, eliminating cross-detection from the closely related SARS-CoV.

To determine the limit of detection, we performed the CRISPR-Cas12-based assay with a target range of 1000 copies/reaction–1 copy/reaction. The concentrations of the RNA sample were adjusted to obtain the desired number of copies/µL of the target. As displayed in Figure 3c, the highest fluorescence signal was observed in the case of 1000 copies/reaction and it decreased as the copy number was reduced to 100, 50, and 10 copies/reaction. The lowest detectable signal was obtained at 10 copies/reaction. In our experimental time range of 30 min, the fluorescence assay conducted with 1 copy/reaction of the target did not show a signal above the control line. We further performed LFAs with the RNA fragments at varying target concentrations. The results showed the clear detection of the SARS-CoV-2 S gene in the range of 1000–50 copies/reaction of the target, with a significant band at the test line. At the level of 10 copies/reaction, a slightly fainter but still distinguishable band was seen compared to 50 copies/reaction. The assay performed with a single copy of the target did not show a significant band at the test line. We additionally performed PCRs with the same RNA samples with varying copy numbers. Unlike fluorescence and LFA assays, we were able to detect signals even at copy numbers as low as 1 copy/reaction, as seen in Figure S1e. We performed similar experiments to verify the limit of detection with the T1 target of the S gene and RNase P, and the results were consistent with that of the assay performed with the T2 target (Figure S1). Based on the results, we concluded that the present SARS-CoV-2 detection assay can detect the virus at a copy number as low as 10 copies/reaction using both fluorescence and naked-eye detection.

### 2.4. Diagnosis of COVID-19 Patients

**Figure 4:**
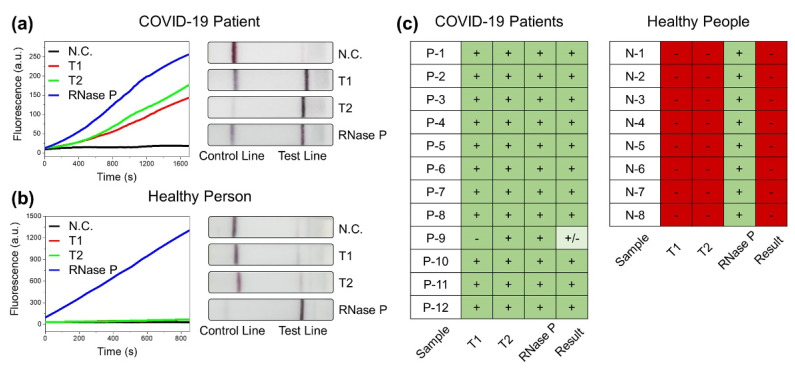
(**a**) Fluorescence and LFA detection results of SARS-CoV-2 in representative COVID-19 patient sample (P-1) using CRISPR-Cas12-based assay. (**b**) Fluorescence and LFA detection results of SARS-CoV-2 in representative healthy person sample (N-1) using CRISPR-Cas12-based assay. Negative control (N.C.) represents the results without samples. (**c**) Interpretation of LFA results for all clinical samples examined. Green represents positive results, red represents negative results, and presumptive positive results are marked with lighter shade of green.

Nasopharyngeal and sputum samples collected from COVID-19 patients were used to examine the diagnostic ability of the CRISPR-Cas12-based assay. The samples were provided by Yonsei University Health Service Center, Severance Hospital, Seoul, Korea after the review and approval by the Institutional Review Board (IRB). For the diagnosis, RNA was extracted using Quick Extract DNA solution. Next, SARS-CoV-2 detection was performed according to the optimized reaction conditions of the CRISPR-Cas12-based assay. In addition, the samples were tested for RNase P detection using POP7 target as an internal positive control. We considered that the sample was COVID-19 positive when the tests showed positive signals for both SARS-CoV-2-specific targets, T1 and T2, and for the POP7 target of RNase P. Fluorescence signal was considered positive or negative in comparison to the background noise. The negative control is the background fluorescence intensity observed in the presence of ssDNA reporter and the absence of any target. The fluorescence signal for each target was considered positive if the continuous increase in fluorescence intensity was observed with time, above the control line, in the experimentally measured time frame. LFA signal was concluded as positive or negative by visual comparison to the intensity of the test line to the negative control. The sample was considered negative when the test was negative for T1 and T2 but positive for the POP7 target. If the test was positive for either T1 or T2 and POP7 positive, it was considered presumptive positive for further validation. Any assay with a negative test for POP7 target was considered invalid. We tested a total of 20 clinical samples using the CRISPR-Cas12-based assay. All 12 samples were detected accurately using the fluorescence assay, with obvious signals for all three targets in the 12 pre-confirmed COVID-19-positive samples (Figure 4a and Figure S2). All the 12 samples were pre-confirmed using PCR with cycle threshold (Ct) values ranging from 14–30 (Table S4). All eight samples collected from healthy individuals tested negative, as no fluorescence signals above the control line were detected for either of the SARS-CoV-2-specific targets, with a positive signal for POP7 target (Figure 4b and Figure S3). The detection of SARS-CoV-2 is up to 100% accurate with fluorescence measurement. Meanwhile, according to the results of LFA performed with 12 COVID-19-positive samples (Figure 4a and Figure S4), 11 samples were positive with clear positive lines for T1, T2, and POP7. One of the samples was considered a presumptive positive as the COVID-19-specific T1 was inconclusive whereas it was positive for T2 and POP7. Of the eight healthy samples assayed using LFA, all eight samples showed negative results for COVID-19 (Figure 4b and Figure S5). This indicates that the developed SARS-CoV-2 detection approach is capable of the diagnosis of COVID-19 patients (Figure 4c).

### 2.5. Versatility of CRISPR-Cas12-Based Assay

**Figure 5:**
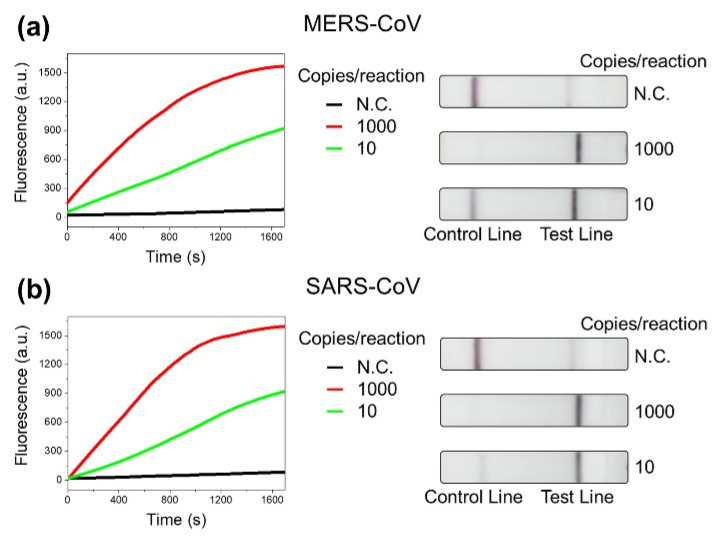
(**a**) Fluorescence and LFA detection results of synthetic gene of MERS-CoV using CRISPR-Cas12-based assay. (**b**) Fluorescence and LFA detection results of synthetic gene of SARS-CoV using CRISPR-Cas12-based assay. N.C. represents the results without samples.

Lastly, we present the extension of our assay in the detection of other human infectious viruses as evidence of its versatility and wide scope for application. Although not on as large a scale as COVID-19, the world has been victim to infectious viruses in the past [25,35]. Some examples include the MERS epidemic of 2012 caused by MERS-CoV and the SARS outbreak of 2002 caused by SARS-CoV [24,36]. We modified the CRISPR-Cas12-based assay to detect the viruses mentioned above. By comparing several sample sequences of respective viral genomes, 20 nucleotide target sequences next to the PAM sequence were selected for MERS-CoV and SARS-CoV, respectively (Figure S5). Using the synthesized gene fragments of the viruses, we demonstrated the detection of MERS-CoV and SARS-CoV. As shown in Figure 5, the gene fragments were detectable at 10 copies/reaction, suggesting the wide applicability of the CRISPR-Cas12-based assay.

## 3. Discussion

The present approach has a few advantages over the conventional PCR method used for SARS-CoV-2 detection. First, it reduces the dependency of the test on the expensive instrumentation required for PCR and facilitates increasing the speed of testing in regions with no access to sophisticated laboratories. Second, our approach significantly reduces the duration of the test, from 4 h for the Centers for Disease Control and Prevention (CDC)-approved PCR method to <120 min. Third, naked-eye detection provides easy-to-analyze results which makes it more public-friendly. Finally, the isothermal method of amplification abolishes the need for thermocycling, which requires special equipment. Several CRISPR-based approaches include the reverse transcription loop-mediated isothermal amplification (RT-LAMP)-based amplification method, which requires the reaction temperature to be maintained at 60–65 °C and has added complexity due to the increased number of primers compared to the RT-RPA method used in this study [29]. The RPA reaction is known to be very tolerant of the reaction temperature and works at temperatures as low as 25 °C, albeit slowly. Previous studies on the temperature dependency of Cas12 proteins have shown the ability of Lbcpf1 to be active even at 25 °C [37]. Given that both amplification and CRISPR-Cas12 recognition are possible at room temperature, our approach has significant potential as it can be optimized as a single-step, room-temperature-operable detection tool. This abolishes the need for instrumentation to maintain the temperature, including incubators, thermocyclers, or heating baths, which allows it to be a simple and portable system.

However, the current system has to undergo further modifications to be used as a POCT system. The system presented here is a preliminary work, where the detection takes place in a multi-step reaction. Although the amplification can be adapted to be carried out at room temperature with optimization, it is necessary to integrate the sample treatment and detection reaction into a single reaction. Currently, the fluorescence read-out is dependent on a lab-set fluorimeter, and it is necessary to integrate a portable fluorescence reader as used in other POCT systems [38]. Additionally, although the LFA read-out can show public-friendly, easy-to-read results without further analysis, the whole reaction from sample preparation to detection is handled in the lab. In order to make our system into a public-friendly POCT system, there is a need for the development of an integrated automated device which can be operated with minimal expertise.

There are several other SARS-CoV-2 detection approaches that have been reported recently that work as a single-step reaction, integrated portable devices for the purpose of POCT, such as CRISPR optical detection of anisotropy (CODA), CRISPR-assisted detection (CASdetec), personal glucose meter–CRISPR assay (PGM-CRISPR), an integrated portable droplet magnetofluidic (DM) device, and CRISPR-fluorescence detection system (CRISPR-FDS) [38,39,40,41,42,43,44]. While these approaches are already functional POCT devices, they are capable of detecting SARS-CoV-2 only. As an assay, our approach is comparable to the abovementioned systems at the level of sensitivity of detection, as shown in Table 1. Additionally, based on sequence comparison from representative candidates, we believe that our assay is capable of detecting other SARS-CoV-2 variants, i.e., alpha, beta, gamma, and delta that were identified in the UK, South Africa, Brazil, and India, respectively (Figure S7) [45,46]. Moreover, our approach also aims at integrating the detection of MERS-CoV and SARS-CoV with SARS-CoV-2 into a single POCT device, which is not available currently. We have selected suitable targets, designed gRNAs and RPA primers for the detection of MERS-CoV and SARS-CoV, and provided preliminary evidence for the detection in this work. Hence, with suitable modifications and development, we believe that our approach has the potential to be a useful POCT device.

## 4. Experimental Section

### 4.1. Materials

All DNA templates used for the synthesis of RNA fragments were purchased from Bioneer Corporation (Daejeon, Korea). RNA gene fragments were produced using an EZ^TM^ MEGA T7 Transcription Kit purchased from Enzynomics (Daejeon, Korea). A Twist Amp^®^ Basic Recombinase Polymerase Amplification Kit used for isothermal amplification was purchased from 2NCBIO Inc. (Daejeon, Korea). Milenia HybriDetect 1 for LFA was purchased from 2NCBIO Inc. RevertAid H Minus Reverse Transcriptase and RiboLock RNase inhibitor were purchased from Thermo Fisher (Waltham, MA, USA). Quick Extract TM DNA Extract Solution was purchased from Lucigen (Waltham, WI, USA). All primers used for RT-RPA reactions (Table S1), gRNA templates (Table S2), and ssDNA reporters were synthesized by Bioneer Corporation.

### 4.2. RNA Gene/gRNA Synthesis

DNA templates for the in vitro transcription were synthesized with the T7 promoter region at 5’ terminal as per the instructions of the Enzynomics MEGA T7 Transcription Kit. Approximately 150–200 ng of template was mixed with 2 µL of each ribonucleotide, 2 µL of T7 buffer, and 2 µL of T7 enzyme in a 20 µL reaction and incubated at 37 °C, overnight, followed by ethanol precipitation.

### 4.3. Amplification of Target Fragments

For RT-RPA reactions, RPA pellets were suspended in 29.5 µL of rehydration buffer, 1 µL of RevertAid H minus reverse transcriptase, 1 µL of RiboLock RNase inhibitor, 2.4 µL of forward primer, 2.4 µL of reverse primer, 1 µL of target RNA, 2.5 µL of magnesium acetate, and 11.2 µL of nuclease-free water. The reaction was incubated at 37 °C for 30 min with frequent mix-spin cycles for better efficiency. The sequence is shown in Table S3.

### 4.4. Cas12 Protein Purification and Cas12/gRNA Complex Formation

Cas12 protein was expressed from a pMAL based vector (pMAL-his-LbCpf1-EC) containing N-terminal maltose binding protein (MBP), 6X His-Tag, and TEV protease cleavage site. Plasmid was a gift from Dr. Dae-sik Kim in Korea Research Institute of Bioscience and Biotechnology. Proteins were purified as described previously [17] and stored in a storage/reaction buffer containing 20 mM Tris-HCl pH 7.5, 100 mM KCl, and 5% glycerol.

Cas12/gRNA complexes were assembled by mixing Cas12 protein with gRNA in a 1:1.5 molar ratio followed by incubation in reaction buffer at 37 °C for 15 min.

### 4.5. CRISPR-Cas12-Based Assay

All fluorescence read-out reactions were carried out in a 96-well microplate. We mixed 2 µL of amplified targets from the RT-RPA reaction with other reaction components to a final concentration of 50 nM of LbCas12a-gRNA complex, 500 nM of ssDNA reporter (FAM-TTATT-BHQ1), and 5 mM of MgCl_2_ in a reaction buffer containing 20 mM Tris-HCl pH 7.5, 100 mM KCl, and 5% glycerol. Fluorescence readings were taken at the parameters λ_exc_ (485 nm) and λ_em_ (535 nm) at 30 s intervals for 30 min at 37 °C in a Victor X3 Multilabel plate reader from PerkinElmer (Waltham, MA, USA).

For negative control, we mixed 50 nM each of LbCas12a-gRNA (T1), LbCas12a-gRNA (T2), and LbCas12a-gRNA (RNase P) with 500 nM. All the three target gRNAs were mixed with 5 mM ssDNA reporter (FAM-TTATT-BHQ1) and 5 mM of MgCl_2_ in a reaction buffer containing 20 mM Tris-HCl pH 7.5, 100 mM KCl, and 5% glycerol. Fluorescence was measured as mentioned above.

For LFA detection, 2 µL of the amplified target was mixed with reaction components to a final concentration of 500 nM of Lbcas12a-gRNA complex, 50 nM of ssDNA reporter (FAM-TTATTATT-Biotin), and 5 mM of MgCl_2_ in the reaction buffer. The reaction was carried out for 45 min at 37 °C. After the completion of the reaction, a lateral flow strip was dipped in the reaction and held upright for 1 min. The results could be visualized by the presence or absence of the test band.

For negative control, we mixed 50 nM each of LbCas12a-gRNA (T1), LbCas12a-gRNA (T2), and LbCas12a-gRNA (RNase P) with 50 nM ssDNA reporter (FAM-TTATTATT-Biotin), and 5 mM of MgCl_2_ in a reaction buffer containing 20 mM Tris-HCl pH 7.5, 100 mM KCl, and 5% glycerol. The reaction was carried out as mentioned above for LFA.

### 4.6. Limit of Detection

RNA targets were obtained from in vitro transcription of synthesized DNA fragments as described in experimental Section 4.2. RNA concentration was measured using NanoDrop (Wilmington, DE, USA). Total RNA copy number was calculated using known concentration and size of the product using the formula, X g/µL RNA/[transcript length in nucleotides × 340]) × 6.022 × 10^23^ = Y molecules/µL. Concentration was adjusted to obtain 10^12^ copies/µL. Then, a series of serial dilutions were performed to obtain a required number of copies. Using the RNA samples, an RT-RPA reaction was performed as described in Section 4.3, followed by CRISPR-Cas12 detection assay as described in Section 4.5.

### 4.7. Clinical Sample Analysis

Nasopharyngeal aspirates and sputum samples from patients were collected with flocked nasopharyngeal swabs and placed into virus transport media (UTM, Copan Diagnostics Inc., Murrieta, CA, USA). RNA was extracted by mixing equal volumes of sample and Quick Extract DNA solution followed by boiling at 95 °C for 10 min. This extract was used directly for the assay. Fluorescence and LFA were carried out as previously described. The protocol for this retrospective study was reviewed and approved by the IRB of Yonsei University Health Service Center, Severance Hospital, Seoul, Korea (IRB approval number: 4-2020-0465).

### 4.8. PCR

RNA samples of desired copy number were prepared as described in Section 4.6. A complementary DNA strand was synthesized using a RevertAid H Minus First Strand cDNA Synthesis Kit purchased from Thermo Fisher. RNA template was mixed with 1 µL of primer, 4 µL of 5× reaction buffer, 1 µL of Ribolock RNase Inhibitor, 2 µL of 10 mM dNTP mix, and 1 µL of RevertAid H Minus Reverse Transcriptase. The reaction was carried out at 42 °C for 60 min.

Products from reverse transcription were used for PCR performed using TOPreal™ qPCR 2× PreMIX (SYBR Green with low ROX) purchased from Enzynomics. Two microliters of the template DNA from reverse transcription was mixed with 10 µL of TOPreal™ qPCR 2× PreMIX, 1 µL of forward primer, 1 µL of reverse primer in a reaction volume of 20 µL. PCR was carried out using a Bio-Rad CFX96 Real-Time PCR instrument (Hercules, CA, USA).

## Figures and Tables

**Table 1:** Comparison of the current approach with previous SARS-CoV-2 detection systems.

Approach	Sensitivity (Copies/µL)	Time (min)	Instrument
[38]	3	20	Compact CRISPR optical detection of anisotropy instrument
[39]	10	30	Portable light-emitting diode
[40]	10	50	Personal glucose meter
[41]	1–10	50	Fluorimeter and lateral flow strips
[42]	0.38	15	Laser diode of smartphone-based fluorescence microscope
[43]	1	30	Portable droplet magnetofluidic device
[29]	10	45	Fluorimeter and lateral flow strips
[44]	42	50	Fluorimeter and lateral flow strips
Current approach	10	60	Fluorimeter and lateral flow strips

## Data Availability

Data are contained within the article or Supplementary Materials.

## Supplementary Materials

The following are available online at https://www.mdpi.com/article/10.3390/bios11090301/s1, Figure S1: Fluorescence and LFA detection results of T1 and RNase P genes using CRISPR-Cas12-based assay and PCR results of T1, T2, and RNase P genes, Figure S2: Fluorescence detection results of SARS-CoV-2 in COVID-19 patient samples using CRISPR-Cas12-based assay, Figure S3: Fluorescence detection results of SARS-CoV-2 in healthy people samples using CRISPR-Cas12-based assay, Figure S4: LFA detection results of SARS-CoV-2 in COVID-19 patient samples using CRISPR-Cas12-based assay, Figure S5: LFA detection results of SARS-CoV-2 in healthy people samples using CRISPR-Cas12-based assay, Figure S6: Sequence alignment of S gene in SARS-CoV and N gene of MERS-CoV, Figure S7: Sequence alignment of S gene in SARS-CoV-2 variants. Regions colored in yellow and skyblue indicate SARS-CoV-2 specific T1 and T2, respectively, next to PAM sequence marked in red. Table S1: Primer list, Table S2: gRNA list, Table S3: dsDNA gene fragments, Table S4: PCR results of clinical samples.

## References

[1] Wang C., Horby P.W., Hayden F.G., Gao G.F. (2020). A novel coronavirus outbreak of global health concern. Lancet.

[2] Zhu N., Zhang D., Wang W., Li X., Yang B., Song J., Zhao X., Huang B., Shi W., Lu R. (2020). A Novel Coronavirus from Patients with Pneumonia in China, 2019. N. Engl. J. Med..

[3] World Health Organization (2020). Coronavirus Disease (COVID-19) Situation Report-209.

[4] Munster V.J., Feldmann F., Williamson B.N., van Doremalen N., Perez-Perez L., Schulz J., Meade-White K., Okumura A., Callison J., Brumbaugh B. (2020). Respiratory disease in rhesus macaques inoculated with SARS-CoV-2. Nature.

[5] Venkatesh S., Memish Z.A. (2004). SARS: The new challenge to international health and travel medicine. East. Mediterr. Health J..

[6] Shultz J.M., Althouse B.M., Baingana F., Cooper J.L., Espinola M., Greene M.C., Espinel Z., McCoy C.B., Mazurik L., Rechkemmer A. (2016). Fear factor: The unseen perils of the Ebola outbreak. Bull. Sci.

[7] Wan Z., Zhang Y., He Z., Liu J., Lan K., Hu Y., Zhang C. (2016). A Melting Curve-Based Multiplex RT-qPCR Assay for Simultaneous Detection of Four Human Coronaviruses. Int. J. Mol. Sci..

[8] Shen M., Zhou Y., Ye J., Abdullah Al-Maskri A.A., Kang Y., Zeng S., Cai S. (2020). Recent advances and perspectives of nucleic acid detection for coronavirus. J. Pharm. Anal..

[9] Tahamtan A., Ardebili A. (2020). Real-time RT-PCR in COVID-19 detection: Issues affecting the results. Expert Rev. Mol. Diagn..

[10] Bachman J. (2013). Reverse-transcription PCR (RT-PCR). Methods Enzym..

[11] Zhang W., Du R.H., Li B., Zheng X.S., Yang X.L., Hu B., Wang Y.Y., Xiao G.F., Yan B., Shi Z.L. (2020). Molecular and serological investigation of 2019-nCoV infected patients: Implication of multiple shedding routes. Emerg. Microbes Infect..

[12] Grifoni A., Sidney J., Zhang Y., Scheuermann R.H., Peters B., Sette A. (2020). A Sequence Homology and Bioinformatic Approach Can Predict Candidate Targets for Immune Responses to SARS-CoV-2. Cell Host Microbe.

[13] Liu G., Rusling J.F. (2021). COVID-19 Antibody Tests and Their Limitations. ACS Sens..

[14] Corman V.M., Haage V.C., Bleicker T., Schmidt M.L., Mühlemann B., Zuchowski M., Jo W.K., Tscheak P., Möncke-Buchner E., Müller M.A. (2021). Comparison of seven commercial SARS-CoV-2 rapid point-of-care antigen tests: A single-centre laboratory evaluation study. Lancet Microbe.

[15] Manghwar H., Lindsey K., Zhang X., Jin S. (2019). CRISPR/Cas System: Recent Advances and Future Prospects for Genome Editing. Trends Plant. Sci..

[16] Gootenberg J.S., Abudayyeh O.O., Lee J.W., Essletzbichler P., Dy A.J., Joung J., Verdine V., Donghia N., Daringer N.M., Freije C.A. (2017). Nucleic acid detection with CRISPR-Cas13a/C2c2. Science.

[17] Chen J.S., Ma E., Harrington L.B., Da Costa M., Tian X., Palefsky J.M., Doudna J.A. (2018). CRISPR-Cas12a target binding unleashes indiscriminate single-stranded DNase activity. Science.

[18] Zaghloul H., El-Shahat M. (2014). Recombinase polymerase amplification as a promising tool in hepatitis C virus diagnosis. World J. Hepatol..

[19] Ding X., Yin K., Li Z., Lalla R.V., Ballesteros E., Sfeir M.M., Liu C. (2020). Ultrasensitive and visual detection of SARS-CoV-2 using all-in-one dual CRISPR-Cas12a assay. Nat. Commun..

[20] (2020). Detection of SARS-CoV-2 with SHERLOCK One-Pot Testing. New Eng. J. Med..

[21] Park J., Hsieh K., Chen L., Kaushik A., Trick A., Wang T.-H. (2021). Digital CRISPR/Cas-Assisted Assay for Rapid and Sensitive Detection of SARS-CoV-2. Adv. Sci..

[22] Yu T., Zhang S., Matei R., Marx W., Beisel C.L., Wei Q. (2021). Coupling smartphone and CRISPR–Cas12a for digital and multiplexed nucleic acid detection. AIChE J..

[23] van Dongen J.E., Berendsen J.T.W., Steenbergen R.D.M., Wolthuis R.M.F., Eijkel J.C.T., Segerink L.I. (2020). Point-of-care CRISPR/Cas nucleic acid detection: Recent advances, challenges and opportunities. Biosens. Bioelectron..

[24] World Health Organization MERS Situation Update. http://www.emro.who.int/pandemic-epidemic-diseases/mers-cov/mers-situation-update-december-2019.html.

[25] LeDuc J.W., Barry M.A. (2004). SARS, the first pandemic of the 21st century. Emerg. Infect. Dis..

[26] Chen B., Tian E.K., He B., Tian L., Han R., Wang S., Xiang Q., Zhang S., El Arnaout T., Cheng W. (2020). Overview of lethal human coronaviruses. Signal. Transduct Target..

[27] Tai W., He L., Zhang X., Pu J., Voronin D., Jiang S., Zhou Y., Du L. (2020). Characterization of the receptor-binding domain (RBD) of 2019 novel coronavirus: Implication for development of RBD protein as a viral attachment inhibitor and vaccine. Cell. Mol. Immunol..

[28] Benvenuto D., Giovanetti M., Ciccozzi A., Spoto S., Angeletti S., Ciccozzi M. (2020). The 2019-new coronavirus epidemic: Evidence for virus evolution. J. Med. Virol..

[29] Broughton J.P., Deng X., Yu G., Fasching C.L., Servellita V., Singh J., Miao X., Streithorst J.A., Granados A., Sotomayor-Gonzalez A. (2020). CRISPR-Cas12-based detection of SARS-CoV-2. Nat. Biotechnol..

[30] Fernandes-Monteiro A.G., Trindade G.F., Yamamura A.M., Moreira O.C., de Paula V.S., Duarte A.C., Britto C., Lima S.M. (2015). New approaches for the standardization and validation of a real-time qPCR assay using TaqMan probes for quantification of yellow fever virus on clinical samples with high quality parameters. Hum. Vaccines Immunother..

[31] Wozniak A., Cerda A., Ibarra-Henríquez C., Sebastian V., Armijo G., Lamig L., Miranda C., Lagos M., Solari S., Guzmán A.M. (2020). A simple RNA preparation method for SARS-CoV-2 detection by RT-qPCR. Sci. Rep..

[32] Swarts D.C., Jinek M. (2019). Mechanistic Insights into the cis- and trans-Acting DNase Activities of Cas12a. Mol. Cell.

[33] Ahn W.C., Park K.H., Bak I.S., Song H.N., An Y., Lee S.J., Jung M., Yoo K.W., Yu D.Y., Kim Y.S. (2019). In vivo genome editing using the Cpf1 ortholog derived from Eubacterium eligens. Sci. Rep..

[34] Wang H., Li X., Li T., Zhang S., Wang L., Wu X., Liu J. (2020). The genetic sequence, origin, and diagnosis of SARS-CoV-2. Eur. J. Clin. Microbiol. Infect. Dis..

[35] Patterson G.E., McIntyre K.M., Clough H.E., Rushton J. (2021). Societal Impacts of Pandemics: Comparing COVID-19 With History to Focus Our Response. Front. Public Health.

[36] World Health Organization (2021). Severe Acute Respiratory Syndrome.

[37] Moreno-Mateos M.A., Fernandez J.P., Rouet R., Vejnar C.E., Lane M.A., Mis E., Khokha M.K., Doudna J.A., Giraldez A.J. (2017). CRISPR-Cpf1 mediates efficient homology-directed repair and temperature-controlled genome editing. Nat. Commun..

[38] Lee C.Y., Degani I., Cheong J., Lee J.H., Choi H.J., Cheon J., Lee H. (2021). Fluorescence polarization system for rapid COVID-19 diagnosis. Biosens. Bioelectron..

[39] Guo L., Sun X., Wang X., Liang C., Jiang H., Gao Q., Dai M., Qu B., Fang S., Mao Y. (2020). SARS-CoV-2 detection with CRISPR diagnostics. Cell Discov..

[40] Huang D., Shi Z., Qian J., Bi K., Fang M., Xu Z. (2021). A CRISPR-Cas12a-derived biosensor enabling portable personal glucose meter readout for quantitative detection of SARS-CoV-2. Biotechnol. Bioeng..

[41] Xiong D., Dai W., Gong J., Li G., Liu N., Wu W., Pan J., Chen C., Jiao Y., Deng H. (2020). Rapid detection of SARS-CoV-2 with CRISPR-Cas12a. PLoS Biol.

[42] Ning B., Yu T., Zhang S., Huang Z., Tian D., Lin Z., Niu A., Golden N., Hensley K., Threeton B. (2021). A smartphone-read ultrasensitive and quantitative saliva test for COVID-19. Sci. Adv..

[43] Chen F.E., Lee P.W., Trick A.Y., Park J.S., Chen L., Shah K., Mostafa H., Carroll K.C., Hsieh K., Wang T.H. (2021). Point-of-care CRISPR-Cas-assisted SARS-CoV-2 detection in an automated and portable droplet magnetofluidic device. Biosens. Bioelectron..

[44] Patchsung M., Jantarug K., Pattama A., Aphicho K., Suraritdechachai S., Meesawat P., Sappakhaw K., Leelahakorn N., Ruenkam T., Wongsatit T. (2020). Clinical validation of a Cas13-based assay for the detection of SARS-CoV-2 RNA. Nat. Biomed. Eng..

[45] World Health Organization (2021). Tracking SARS-CoV-2 Variants.

[46] Bates T.A., Leier H.C., Lyski Z.L., McBride S.K., Coulter F.J., Weinstein J.B., Goodman J.R., Lu Z., Siegel S.A.R., Sullivan P. (2021). Neutralization of SARS-CoV-2 variants by convalescent and vaccinated serum. medRxiv.

