# Supplementary material for: Detection of Infectious Viruses Using CRISPR-Cas12-Based Assay
Source: Biosensors (Basel). 2021 Aug 28;11(9):301. doi: 10.3390/bios11090301 (PMC8468381; doi:10.3390/bios11090301)
Supplement: Supplementary file 1 [file biosensors-11-00301-s001.zip › biosensors-1335222-supplementary.pdf]

Supplementary Materials

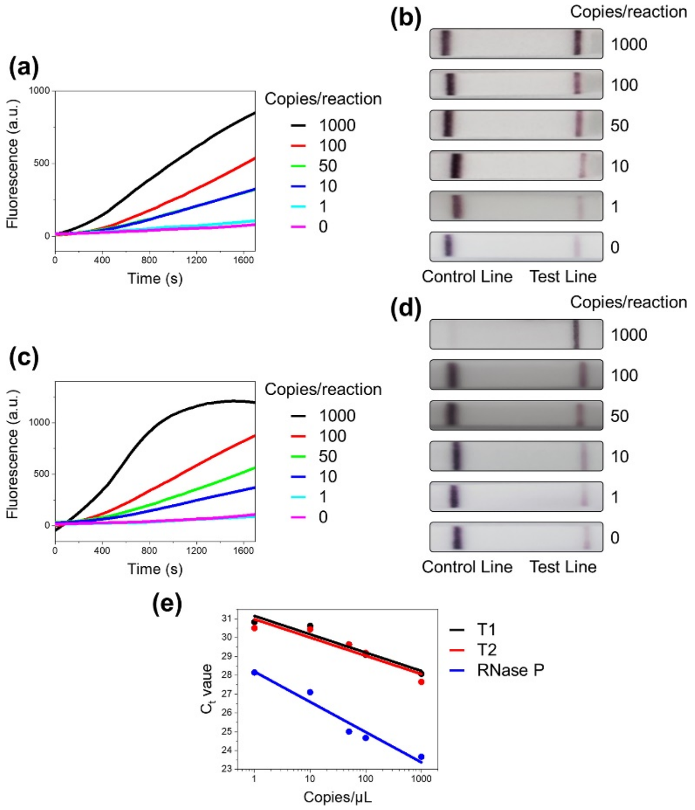

Figure S1. (a) Fluorescence and (b) LFA detection results of synthetic S gene of SARS-CoV-2 (T1) using CRISPR-Cas12-based assay. (c) Fluorescence and (d) LFA detection results of synthetic RNase P gene using CRISPR-Cas12-based assay. Copy number of T2 and RNase P was varied from 1000 to 1 copy/reaction. (e) PCR results of synthetic S gene of SARS-CoV-2 (T1 and T2) and RNase P gene. Copy numbers were varied from 1000 to 1 copy/reaction.

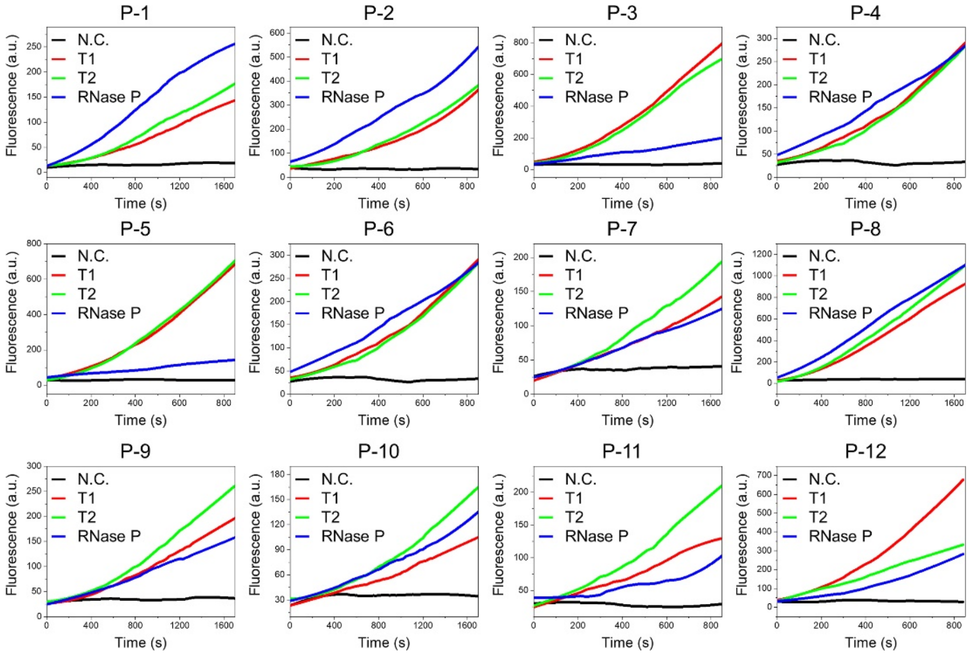

Figure S2. Fluorescence detection results of SARS-CoV-2 in COVID-19 patient samples using CRISPR-Cas12-based assay.

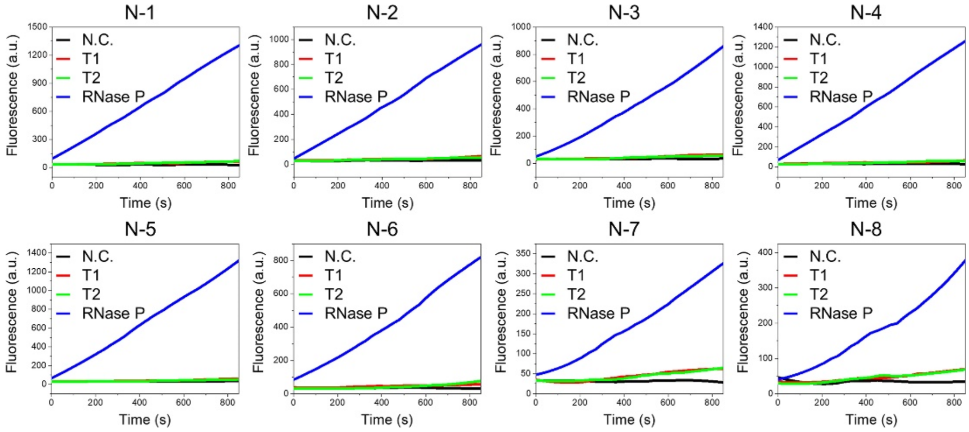

Figure S3. Fluorescence detection results of SARS-CoV-2 in healthy people samples using CRISPR-Cas12-based assay.

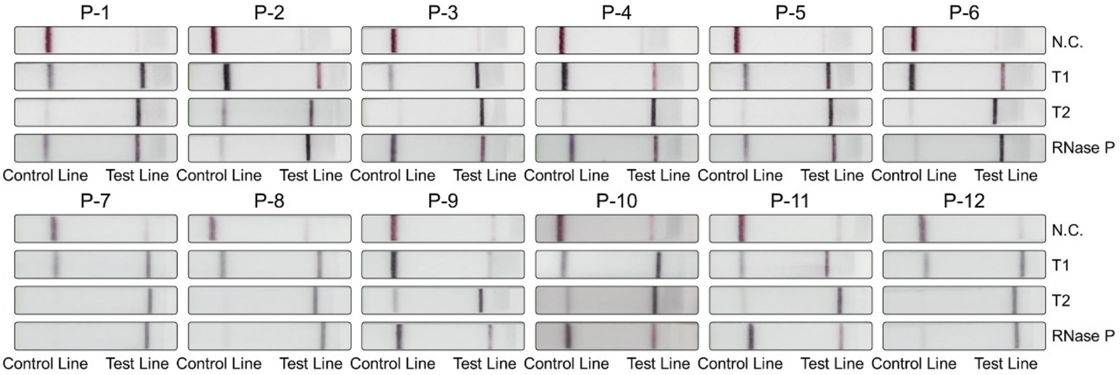

Figure S4. LFA detection results of SARS-CoV-2 in COVID-19 patient samples using CRISPR-Cas12-based assay.

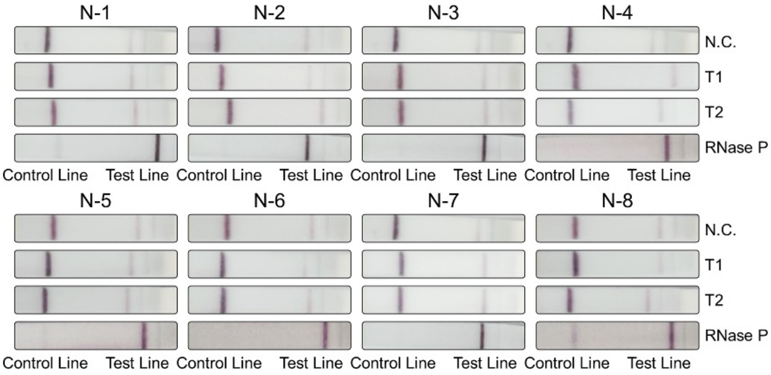

Figure S5. LFA detection results of SARS-CoV-2 in healthy people samples using CRISPR-Cas12-based assay.

```
(a)
                      23451                                                               23520
AY278488 SARS-COV     gctggcatttgtgctagttaccatacagtttctttattacgtagtactagccaaaaatctattgtggctt
AY502925 SARS-CoV     gctggcatttgtgctagttaccatacagtttctttattacgtagtactagccaaaaatctattgtggctt
AY686863 SARS-CoV     gctggcatttgtgctagttaccatacagtttctttcattacgtagtactagccaaaaatctattgtggctt
(b)
                      29191                                                               29260
JX869059 MERS-CoV     taggaggtgatctactttaccttgatcttctgaacagactacaagcccttgagtctggcaaagtaaagca
KC667074 MERS-COV     taggaggtgatctactttaccttgatcttctgaacagactacaagcccttgagtctggcaaagtaaagca
KF192507 MERS-CoV     taggaggtgatctactttaccttgatcttctgaacagactacaagcccttgagtctggcaaagtaaagca
```

Figure S6. Sequence alignment of (a) S gene in SARS-CoV and (b) N gene of MERS-CoV. Regions colored in magenta are the selected target regions for the detection.

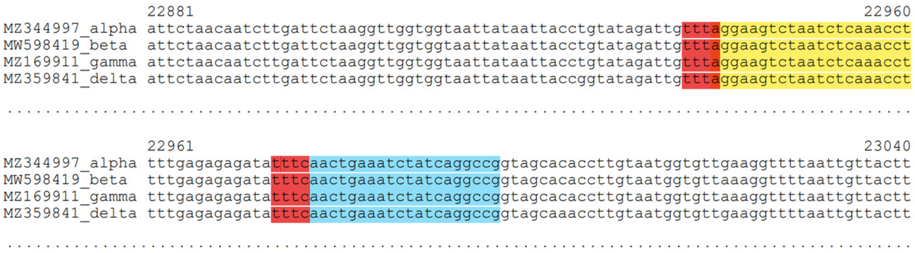

Figure S7. Sequence alignment of S gene in SARS-CoV-2 variants. Regions colored in yellow and skyblue indicate SARS-CoV-2 specific T1 and T2, respectively, next to PAM sequence marked in red.

Table S1. Primer list.

**Name**	**Sequence**
SARS-CoV-2 S FP	ACAGGCTGCGTTATAGCTTGGAATTCTAAC
SARS-CoV-2 S RP	CCTTCAACACCATTACAAGGTGTGCTACCG
SARS-CoV S FP	TGAGTGCGACATTCCTATTGGAGCTGGCAT
SARS-CoV S RP	GTAGGTATAGCAATGGTGTTATTAGAG-TAA
MERS-CoV N FP	GTTCACAAGGTTCAAGATCAG-GAAACTCTA
MERS-CoV N RP	TTCTTAGTGATTACTTTTGGCTGCGATTGC
RNase P FP	GTGGAGCTTGTTGATGAGCTG-GAGCCAGAG
RNase P RP	TCCGAGGTCCAGTACTCAGCATGCGAA-GAG

Table S2. gRNA list.

Name	Sequence
SARS-CoV-2 T1 gRNA	UAAUUUCUACUAAGUGUAGAUGGAA-GUCUAAUCUCAAACCU
SARS-CoV-2 G2 gRNA	UAAUUUCUACUAAGUGUAGAU-AACUGAAAUCUAUCAGGCCG
RNase P gRNA	UAAUUUCUACUAAGUGUAGAUAAUUAC-UUGGGUGUGACCCU
Ascpf1 T1 gRNA	UAAUUUCUACUUUGUAGAUGGAAGUCU-AAUCUCAAACCU
Eecpf1 T1 gRNA	UAAUUUCUACUUUGUAGAUGGAAGUCU-AAUCUCAAACCU
MERS-CoV N gRNA	UAAUUUCUACUAAGUGUAGAC-CUUGAUCUUCUGAACAGAC
SARS-CoV S gRNA	UAAUUUCUACUAAGUGUAGAU-UUAUUACGUAGUACUAGCC

Table S3. dsDNA gene fragments.

Name	Sequence
SARS-CoV-2 S gene	GAAATTAATACGACTCACTATAGGGATGATTTTACAGGCTGCGTTATAGCTT-<br>GGAATTCTAACAATCTTGATTCTAAGGTTGGTGGTAATTATAATTACCTGTAT<br>AGATTGTTTAGGAAGTCTAATCTCAAACCTTTTGAGAGAGA-<br>TATTTCAACTGAAATCTATCAGGCCGGTAGCACACCTTGTAATGGTGTTGAA<br>GGTTTTAATTGTTACT
SARS-CoV S gene	GAAATTAATACGACTCACTATAGGGCGACACTTCTTATGAGTGCGACATTCC-<br>TATTGGAGCTGGCATTTGTGCTAGTTACCATACAGTTTCTTTATTACGTAGTAC<br>TAGCCAAAAATCTATTGTGGCTTATACTATGTCTTTAGGTGCTGA-<br>TAGTTCAATT-<br>GCTTACTCTAATAACACCATTGCTATACCTACTAACTTTTCAATTAG
MERS-CoV N gene	GAAATTAATACGACTCACTATAGGGCAGATCTAGTTCACAAGGTTCAA-<br>GATCAGGAAACTC-<br>TACCCGCGGCACTTCTCCAGGTCCATCTGGAATCGGAGCAGTAGGAGGTGAT<br>CTACTTTACCTTGATCTTCTGAACAGACTACAAGCCCTTGAGTCTGG-<br>CAAAGTAAAGCAATCGCAGCCAAAAGTAATCACTAAGAAAGATGCTGCT

Table S4. PCR results of clinical samples.

Sample number	Sample type	Ct value
1	Nasopharyngeal aspirates	20.85
2	Sputum	24.67
3	Nasopharyngeal aspirates	14.41
4	Nasopharyngeal aspirates	29.75
5	Sputum	20.66
6	Nasopharyngeal aspirates	18.91
7	Sputum	17.86
8	Nasopharyngeal aspirates	19.61
9	Sputum	23.27
10	Nasopharyngeal aspirates	25.93
11	Sputum	23.99
12	Sputum	22.43
